# Levelling up health in the early years: A cost-analysis of infant feeding and healthcare

**DOI:** 10.1371/journal.pone.0300267

**Published:** 2024-05-22

**Authors:** Omotomilola Ajetunmobi, Emma McIntosh, Diane Stockton, David Tappin, Bruce Whyte

**Affiliations:** 1 Public Health Scotland (Formerly Information Services Division, NHS National Services Scotland), Edinburgh, Scotland, United Kingdom; 2 Health Economics and Health Technology Assessment, School of Health and Wellbeing, University of Glasgow, Glasgow, Scotland, United Kingdom; 3 Public Health Scotland (Formerly NHS Health Scotland), Edinburgh, Scotland, United Kingdom; 4 Child Health, School of Medicine, Dentistry & Nursing, University of Glasgow, Glasgow, United Kingdom; 5 Glasgow Centre for Population Health, Glasgow, Scotland, United Kingdom; The University of Sydney, AUSTRALIA

## Abstract

**Background:**

Although breastfeeding is recommended as the optimal form of nutrition in the first six months, it is not sustained as the predominant mode of feeding infants in Scotland. This study estimated the impact of infant feeding choices on primary and secondary healthcare service costs in a 13-year birth cohort.

**Method:**

Using linked administrative datasets, in a retrospective cohort design of 502,948 singletons born in Scotland between 1997 and 2009, we estimated the cost of GP consultations and hospital admissions by area deprivation and mode of infant feeding up to 6–8 weeks for ten common childhood conditions from birth to 27 months. Additionally, we calculated the potential healthcare savings if all infants in the cohort had been exclusively breastfed at 6–8 weeks. Discounting of 1.5% was applied following current health economic conventions and 2009/10 used as the base year.

**Results:**

Over the study period, the estimated cost of hospital admissions in the cohort was £111 million and £2 million for the 2% subset of the cohort with primary care records. Within each quintile of deprivation, exclusively breastfed infants used fewer healthcare services and incurred lower costs compared to infants fed (any) formula milk. At least £10 million of healthcare costs may have been avoided if formula-fed infants had been exclusively breastfed within the first 6–8 weeks of birth.

**Conclusions:**

This study using a representative birth cohort demonstrates how breastmilk can promote equitable child health by reducing childhood illness and healthcare utilisation in the early years.

## Introduction

There is little debate about the importance of breastfeeding for promoting development and preventing disease in the early years [1–8]. Breast milk, which is accessible, natural, and proven to be cost effective ([8–20], is described as a ‘smart investment’ [9] and recommended as the exclusive method of infant feeding in the first six months after birth [21]. Nevertheless, in Scotland and the United Kingdom as a whole, breastfeeding rates fall progressively from birth with persistent patterns of formula feeding in the more deprived areas contributing to inequalities in early childhood [22–26] despite supportive government policy [27].

Government policies to promote child health have made little progress; improvements where present are often unevenly distributed and have failed to reduce health inequalities [28,29]. More evidence of the differential effectiveness of interventions is needed [28], to support policy decisions and efficient resource allocation, which is difficult to demonstrate in paediatric healthcare [29,30].

Using linked administrative data for a thirteen-year Scottish birth cohort, we estimate the direct healthcare costs (and potential savings) of infant feeding choices, across socio-economic gradients for common childhood illnesses.

## Methods

### Study population

The study population comprised 502,948 singletons born in Scotland between 1997 and 2009 with valid infant feeding records at the 6-8-week review. As reported in a previous publication, the study population excluded multiple births (3%), infants with invalid feeding records (8%), those admitted to special facilities e.g., high dependency units or diagnosed with a congenital disorder (4%) or conditions that originated during the perinatal period (1.7%) and non-Scottish residents (1.7%)—[5,23]. In addition, the records of four infants with invalid postcode records (from which deprivation quintile areas were derived), were excluded from this study.

### Data sources

Retrospective data extracts from routine and administrative data systems, held by Public Health Scotland (formerly the Information Services Division, NHS National Services Scotland), were linked in two phases using probability matching algorithms [23] and a unique identifier [5]. The linked extracts comprised records of births, deaths, migration, maternity, infant and hospital admission episodes (up to March 2012) [5] and GP primary care consultations (April 2003 to March 2012), which were anonymised before analyses. The aggregate costs of community prescriptions in 2009 for children (by age in years) was also obtained from Public Health Scotland.

The subset of infants with primary care records (hereafter referred to as the primary care subset), comprised 2% of the infants in the full 13-year birth cohort. These records were obtained from 34 GP practices consistently involved in the national Practice Team Information (PTI) data system. The PTI data was a 6% sample of general practices, reasonably representative of the Scottish population with regards to age, gender and deprivation [31], which commenced in 2003 and ended in 2009.

Approval for the study was granted by the Privacy Advisory Committee NHS National Services Scotland (now known as the Public Benefit and Privacy Panel). Each GP practice included in the study also granted permission to use their records, which were anonymised before release for analysis in the study. Further ethical approval was not required.

### Definitions

#### Infant feeding

This was the predominant mode of feeding recorded on the day preceding the 6–8 week health visitor review visit, described as either exclusive breastfeeding, formula feeding or mixed (breast and formula) feeding.

#### Childhood illnesses

The study outcomes comprised childhood illnesses associated with infant feeding either a priori in the literature or commonly reported in the cohort [4]; defined by the WHO International Classification of Disease—ICD10 codes for hospital admissions and Read codes–Scottish Version 2 for GP consultations. The childhood illnesses were gastrointestinal, respiratory (lower and upper) and urinary tract infections, otitis media, asthma, eczema, diabetes, dental caries and fevers (See Table A in S1 Appendix in S3 File).

#### Follow up

For all children in the cohort, the cut-off of 27 months was the maximum period of uniform follow-up. Costs were analysed over two follow-up periods to represent the time before and after the recommended duration of exclusive breastfeeding [4], i.e., 0–6 months and 7–27 months.

#### Deprivation

The Scottish Index of Multiple Deprivation (SIMD) is an area-based composite measure of relative deprivation calculated for data zones (small area geographical units) made up from indicators across seven domains [32]. For this study, deprivation quintiles were calculated (using the 2006 version of SIMD) by mapping the postcode of parental residence of each birth record to a data zone, from which a relative deprivation score from ‘most deprived’ to ‘least deprived’ was assigned.

#### Population attributable fractions (PAFs)

The PAFs are used to quantify the potential outcomes that may have been avoided in the absence of a risk factor(s), ceteris paribus. We used it to estimate the number (and costs) of hospital admissions or GP consultations that may have been avoided if formula feeding was absent in the cohort and all children had been exclusively breastfed to 6–8 weeks. The PAFs were derived from the adjusted relative risk associated with different modes of infant feeding and reported childhood illnesses i.e., adjusted for demographic, infant, parental and socioeconomic characteristics of the cohort) for both hospital admission [5] and GP consultations.

#### Cost analyses

The healthcare costs were calculated separately for hospital admissions and GP consultations and adjusted for inflation using the Hospital and Community Health Service (HCHS) index pay and price changes with 2009/2010 as the base year [33]. An annual discount rate of 1.5% was applied to costs, as recommended by the National Institute for Health and Economic Care Excellence [34]) for public health economic evaluations. For the sensitivity analyses, a discount rate of 3.5% was applied. Analyses were conducted using SPSS vs21 and STATA vs11/ vs13.

#### Hospital costs

The direct costs of hospital care were estimated from 2009/10 Health Resource Groupings (HRGs) for the Scottish National Tariff [35]. The HRG costs were derived from a combination of Scottish hospital costs/activity and comparable English HRG relative resource weights that account for the type of admission (emergency or elective) and complexity of case mix of each hospital event based on the primary diagnosis on admission.

Hospital admissions with multiple HRG codes were assigned the code consistent with Scottish National Tariff guidance [35]. Admissions without an HRG code (due to missing/unmatched speciality code: <1%) were assigned the average (median) costs of hospital admission for the medical paediatric speciality by type of admission within the same year. Length of hospital stay was measured in days; day-case admissions were counted as 0.5 days.

#### GP costs

The cost of each consultation for any of the selected conditions was estimated using the average cost of consultation by a General Practitioner based on the type of consultation [36], regardless of complexity or length of consultation. The average cost of community prescriptions for 2009/10 was applied to each consultation based on patient age in years (Box 1).

**Box 1. Summary of unit costs for primary care GP consultations**

**Table:** 

Description of Costs	Source/Details/Assumptions
GP consultations (varied by length and type of consultation)	Unit Costs for Health and Social Care 2010 [36] Surgery consultation (11 mins): £36 Telephone consultation: £22 Out of hours (face to face)– 17 mins): £53 Administrative: £36 Home visit: £120
Community prescriptions (average costs of medicines prescribed in the community for children in 2009/10 by age)	Prescribing Information System (PIS) request. Public Health Scotland (previously ISD Scotland, NHS National Services Scotland). Infants aged less than 1 year: £9.07 Infants aged 1–2 years: £8.36

Sources: Unit costs for Health and Social Care 2010 [36] and Public Health Scotland

For this study, we assumed that each consultation reported in the primary care subset required medication, based on evidence of relatively high prescribing rates for infections in this age group [37].

## Results

### Hospital admission costs (secondary care)

Of the 502,944 infants included in the analyses, 27% were exclusively breastfed, 9% mixed fed and 64% formula fed at the 6–8 week review. There was also a socioeconomic gradient in mode of infant feeding; the proportion of exclusively breastfed infants ranged from 45% in the least deprived areas to 13% in the most deprived areas (i.e., SIMD 1: 45%, SIMD 2: 38%, SIMD 3: 29%, SIMD 4: 21% and SIMD 5: 13%). Most of the costs were for unplanned events (93%). Hospital admissions for infections predominated—lower (40%) and upper (17%) respiratory tract infections, gastrointestinal infections (20%) and urinary tract infections (5%). A full description of hospital admissions in the cohort, including estimated population attributable fractions has been reported elsewhere [5].

By six months, 5% (n = 27,542) of the cohort had been admitted to hospital for at least one of the selected conditions and 14% (n = 72,218) within 7–27 months.

The total cost of hospital care for the entire cohort over the full study period of 27 months was £111million approximately £221 per child, comprising of £33 million within the first six months of birth (average: £66 per child) and £78 million within 7–27 months, an average of £155 per child (Table 1).

**Table 1:** Hospital admission costs and infant feeding at 6–8 weeks for the 1997–2009 birth cohort.

Mode of feeding	SIMD Quintile	Infants (total*)	0–6 months			7–27 months			0–27 months		
			Adm. rate	Costs	Average (mean) costs	Adm. rate	Costs	Average (mean) costs	Adm. Rate	Costs	Average (mean) costs
Exclusive Breast-feeding	SIMD 5_ Least deprived	39638	111.7	£1,447,930	£37	127.0	£4,703,622	£119	128.9	£6,151,551	£155
	SIMD 4	32519	117.6	£1,366,935	£42	127.3	£4,112,618	£126	131.0	£5,479,553	£169
	SIMD 3	25499	116.3	£1,054,933	£41	132.5	£3,216,957	£126	134.8	£4,271,890	£168
	SIMD 2	21600	116.0	£987,149	£46	133.2	£2,865,412	£133	135.7	£3,852,560	£178
	SIMD 1_Most deprived	18134	113.9	£927,578	£51	133.3	£2,457,717	£136	135.4	£3,385,295	£187
	Total	137,390	115.0	£5,784,524	£42	130.0	£17,356,326	£126	132.6	£23,140,849	£168
Mixed feeding	SIMD 5_ Least deprived	10855	115.2	£579,598	£53	129.1	£1,424,796	£131	132.7	2,004,394	£185
	SIMD 4	9484	115.5	£406,507	£43	131.9	£1,414,267	£149	134.4	1,820,774	£192
	SIMD 3	7889	117.3	£389,858	£49	130.6	£1,144,951	£145	133.7	1,534,809	£195
	SIMD 2	7986	115.6	£434,679	£54	131.1	£1,128,235	£141	133.6	1,562,914	£196
	SIMD 1_Most deprived	7937	117.1	£496,935	£63	138.8	£1,187,096	£150	140.9	1,684,031	£212
	Total	44,151	116.1	£2,307,577	£52	134.0	£6,299,345	£143	140.0	£8,606,922	£195
Formula feeding	SIMD 5_ Least deprived	37181	116.3	£2,133,607	£57	130.4	£5,413,963	£146	134.5	£7,547,570	£203
	SIMD 4	44571	118.6	£2,875,180	£65	133.3	£7,362,381	£165	137.6	10,237,561	£230
	SIMD 3	53970	119.7	£4,049,214	£75	134.0	£8,864,452	£164	139.6	12,913,666	£239
	SIMD 2	75001	120.5	£6,156,718	£82	134.4	£13,491,862	£180	141.1	19,648,580	£262
	SIMD 1_Most deprived	110680	121.5	£10,035,009	£91	135.0	£19,184,937	£173	141.9	29,219,946	£264
	Total	321403	120.2	£25,249,728	£79	132.0	£54,317,595	£169	134.9	£79,567,324	£248
**TOTAL**		**502944**	**119.0**	**£33,341,829**	**£66**	**133.0**	**£77,973,266**	**£155**	**138.0**	**£111,315,095**	**£221**

Source: Public Health Scotland *Excludes 4 infants missing SIMD records due to unmatched postcodes.

**Fig 1:**
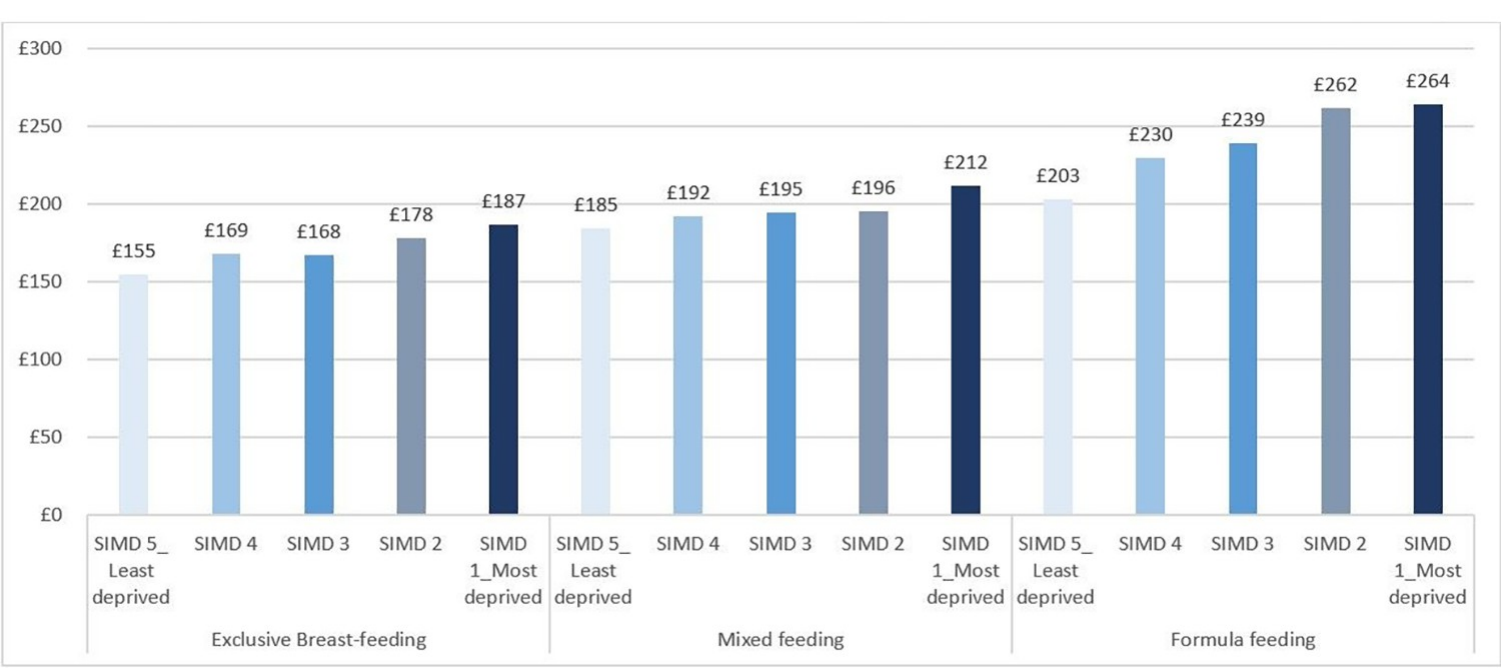
Average admission costs (0–27 months) by mode of infant feeding and deprivation area. Source: Public Health Scotland. Figure includes breakdown of hospital admission costs for common childhood illnesses by mode of infant feeding and deprivation quintile. The childhood illnesses include: gastrointestinal, respiratory (lower and upper) and urinary tract infections, otitis media, asthma, eczema, diabetes, dental caries and fevers.

There was a gradient relating to mode of infant feeding and deprivation quintile in the pattern of hospital admissions and estimated costs. For example, in the first six months, average cost of hospital admission per child was £42 for exclusively breastfed, £52 for mixed fed and £79 for formula fed infants. Formula fed infants also had higher average costs of hospital care than exclusively breastfed infants within and across each deprivation quintile, including exclusively breastfed infants in the most deprived quintile (Fig 1).

If formula fed infants had been exclusively breastfed until the 6–8 week review, about 10% of the hospital admission costs could have been avoided (Table 3). These potential savings were also evident across the deprivation quintiles. For example, in terms of hospital costs within the first six months, the potential savings–if all infants had been exclusively breast fed—ranged from 3% in the least deprived quintile to 10% in the most deprived quintile (not shown).

#### GP consultation costs (primary care)

The primary care subset comprised 11,282 children. The infant feeding characteristics of the subset was similar to the full cohort. However, compared to the full cohort, there were fewer infants in the primary care subset from large urban settlements (primary care subset 22% compared to full cohort: 40%) and from relatively deprived areas i.e., SIMD 1–2 (primary care subset: 39% compared to full Cohort: 45%).

Overall, the majority of the GP consultations reported were surgery visits (91%) followed by out-of-hours care (8%), home visits and telephone consultations (1%). Noting that one GP consultation could include a record of several distinct complaints, the main conditions recorded were respiratory infections, which made up 31% of the consultations, gastrointestinal infections (6.3%), eczema (5.1%), otitis media (4.5%). Other complaints such as urinary tract infections, asthma, diabetes, fevers each comprised less than one percent of the recorded reasons for consultations amongst infants in the primary care subset. There was no record of consultations for dental conditions.

Within six months of birth 58% (n = 5,468) of the infants reported a GP consultation for one or more of the ill health conditions of interest. Exclusively breastfed infants had relatively fewer consultations per child i.e., median number of GP consultations per child (1.72 *95% CI*: 1.66–1.79) compared to mixed fed (1.85, *95% CI*: 1.74–1.98) and formula fed infants (1.92 *95% CI*: 1.88–1.94). By six months, there was a greater relative risk of GP consultations for the selected ill-health conditions in the primary care subset amongst mixed fed infants and formula fed infants (Mixed fed HR 1.14 [CI: 1.02–1.29]; PAF: 1%); Formula fed HR: 1.33 [CI: 1.22–1.41]; PAF: 16%) compared to the exclusively breastfed infants (reference group).

Within 7–27 months, 81% of the infants in the primary care subset reported a GP consultation for one of the ill health conditions studied (n = 9,194). The median number of GP consultations per child was one and did not vary by infant feeding or deprivation quintile. The rates of children consulting the GP among exclusively breastfed infants was 803 per 1000 (95%CI: 755–851) compared to 821 per 1000 (95% CI: 792–850) amongst mixed fed infants and 819 per 1000 (95% CI: 741–896) among formula fed infants. The relative risk of GP consultations was also greater in formula fed infants at 7–27 months (mixed fed infants HR: 1.04 [CI: 0.96–1.13] PAF: 0% and formula fed HR: 1.14 [CI; 1.08–1.19]; PAF: 8% respectively) compared to infants that reported exclusive breastfeeding.

Overall, the total GP consultation costs in the primary care subset was £2,114,980; an average of £187 per child; community prescriptions comprised about a fifth of the total costs (Table 2). Mixed and formula fed infants had greater average costs of £178 and £197 respectively compared to exclusively breastfed infants (£166); a similar pattern was observed at both 0–6 months and 7–27 months and across each of the deprivation quintiles (Table 2). Infants in the least deprived quintile also had relatively greater costs of healthcare compared to infants in the other deprivation quintiles in the 7–27 months period and overall. If all infants in the primary care subset had been exclusively breastfed until the 6–8 week review, 7% of the costs may have been avoided (Table 3).

**Table 2:** Costs of GP consultations by deprivation and infant feeding in the primary care subset.

Mode of feeding at 6–8 weeks	SIMD Quintile	Infants	0–6 months					7-27months				0–27 months	
			GP costs	Prescription costs	Total costs	Mean costs		GP costs	Prescription costs	Total costs	Mean costs	Total Costs	Mean costs
Exclusive Breast-feeding	SIMD 5_ Least deprived	657	£16,619	£4,021	£20,641	£31		£80,237	£18,241	£98,478	£150	£119,119	£181.31
	SIMD 4	700	£17,469	£4,257	£21,726	£31		£75,740	£17,491	£93,231	£133	£114,957	£164.22
	SIMD 3	717	£18,402	£4,457	£22,859	£32		£77,038	£17,355	£94,393	£132	£117,252	£163.53
	SIMD 2	456	£11,496	£2,800	£14,297	£31		£47,401	£10,701	£58,102	£127	£72,399	£158.77
	SIMD 1_ Most deprived	339	£8,215	£1,961	£10,176	£30		£33,905	£7,525	£41,430	£122	£51,606	£152.23
	Total	2869	£72,202	£17,497	£89,698	£31		£314,321	£71,313	£385,634	£134	£475,332	£165.68
Mixed feeding	SIMD 5_ Least deprived	200	£6,640	£1,577	£8,216	£41		£24,057	£5,454	£29,512	£148	£37,728	£188.64
	SIMD 4	236	£6,513	£1,529	£8,042	£34		£26,515	£5,914	£32,429	£137	£40,471	£171.49
	SIMD 3	240	£7,741	£1,853	£9,594	£40		£27,007	£6,036	£33,043	£138	£42,637	£177.65
	SIMD 2	205	£6,094	£1,462	£7,557	£37		£24,248	£5,437	£29,685	£145	£37,242	£181.67
	SIMD 1_ Most deprived	138	£3,972	£930	£4,902	£36		£14,868	£3,275	£18,143	£131	£23,045	£166.99
	Total	1019	£30,960	£7,351	£38,311	£38		£116,696	£26,116	£142,812	£140	£181,123	£177.75
Formula feeding	SIMD 5_ Least deprived	759	£25,863	£6,257	£32,120	£42		£101,866	£22,884	£124,750	£164	£156,870	£206.68
	SIMD 4	933	£33,679	£8,126	£41,805	£45		£121,904	£27,636	£149,539	£160	£191,344	£205.08
	SIMD 3	1775	£61,810	£14,591	£76,401	£43		£219,109	£49,052	£268,161	£151	£344,562	£194.12
	SIMD 2	1839	£65,237	£15,298	£80,536	£44		£226,347	£50,203	£276,550	£150	£357,086	£194.17
	SIMD 1_ Most deprived	2088	£80,871	£18,939	£99,810	£48		£252,982	£55,872	£308,854	£148	£408,664	£195.72
	Total	7394	£267,461	£63,211	£330,671	£45		£922,207	£205,647	£1,127,854	£153	£197.26	£197.26
Grand total		**11,282**	**£370,623**	**£88,059**	**£458,681**	**£41**		**£1,353,224**	**£303,076**	**£1,656,299**	**£147**	**£2,114,980**	**£187.46**

Source: Public Health Scotland (PTI data, Community Prescribing).

**Table 3:** Summary of healthcare costs and potential savings.

Healthcare setting	Mode of infant feeding at 6–8 weeks	0–6 months			7–27 months			0–27 months		
		Total cost	Mean cost	Attributable cost	Total cost	Mean cost	Attributable cost	Total cost	Mean cost	Total attributable costs
Hospital admission (full cohort n = 502,944)	Exclusive breastfeeding	£5,784,524	£42	£0 (Reference)	£17,356,326	£126	£0 (Reference)	£23,140,850	£168	£0 (Reference)
	Mixed feeding	£2,307,577	£52	£23,076	£6,299,345	£143	£62,993	£8,606,922	£195	£86,069
	Formula feeding	£25,249,728	£79	£4,544,951	£54,317,595	£169	£5,431,760	£79,567,323	£248	£9,976,711
	**Hospital Total**	**£33,341,829**	**£66**	**£4,568,027**	**£77,973,266**	**£155**	**£5,494,753**	**£111,315,095**	**£221**	**£10,062,780**
GP Consultation (primary care subset n = 11,282)	Exclusive breastfeeding	£89,698	£31	£0 (Reference)	£385,634	£134	£0 (Reference)	£475,332	£166	£0 (Reference)
	Mixed feeding	£38,311	£38	£383	£142,812	£140	£0	£181,123	£178	£383
	Formula feeding	£330,671	£45	£52,907	£1,127,854	£153	£90,228	£1,458,525	£197	£143,136
	**GP Total**	**£458,681**	**£41**	**£53,290**	**£1,656,299**	**£147**	**£90,228**	**£2,114,979**	**£187**	**£143,519**

Source: Public Health Scotland.

## Discussion

In the UK, universal healthcare provision is free at the point of use and costs are borne by the National Health Service (NHS). By comparing ‘alternative courses of action, in terms of both the costs and the consequences’ [23,30], our study demonstrates the health and economic value of breastfeeding in the early years i.e., relatively lower healthcare use/costs of the order of £100 per infant exclusively breastfed to 6 weeks postnatal age. Furthermore, the potential savings that may have accrued if bottle-fed infants had been exclusively breastfed was evident across all deprivation quintiles and greatest in the most deprived quintile. This suggests that increasing breastfeeding rates in the most deprived areas could contribute to the narrowing of inequalities in the early years.

Several studies have explored the economic value of breastmilk for infants, mothers and the society [8–20]. Our study confirms the reported patterns of healthcare use [3,4,8], econometric costs projections and potential savings of increased breastfeeding duration and rates [12–16]. For example, the UK study by Pokhrel et al [15] estimated annual healthcare savings of at least £11 million, in the treatment of childhood gastrointestinal and lower respiratory tract infections and acute otitis media, if breastfeeding rates within the first week of birth were maintained for up to four months. Our study, which included these common childhood infectious conditions observed from birth to 27 months, found potential savings of at least £10 million i.e., had all infants in the cohort been exclusively breast-fed up to the 6–8-week review and showed greater impacts amongst infants in the most deprived quintile; a finding that is still relevant due the persistent social gradients in infant/child health and nutrition [22,24,25]. Allowing for inflation [38], in 2023 the estimated hospital admission costs would have been £170 million for the Scottish cohort and £3.2 million in the primary care subset, all other factors remaining the same.

Furthermore, our study provides a framework for evaluating the potential impact of interventions on child health inequalities that could be used to inform future policy decisions that underpin interventions [11], such as the NOSH study [39]. The NOSH study, a large cluster randomised trial that found positive impacts of financial incentives to women in areas with low breastfeeding rates, was limited by the unavailability of health service utilization data [39].

To be clear, no form of infant feeding is without cost [40,41]. Moreover, the personal and societal costs of infant feeding, which did not form part of this study, could be substantial. For example, if the personal cost of purchasing formula milk for 6–8 weeks was included for this cohort, an extra £26m could have been added to the costs of formula feeding (based on average costs for leading brands in 2009 at £8.99 per 900g serving for 7 weeks). For breastfeeding mothers, the costs of other feeding accessories such as feeding cloths, milk expressing pump, bottles, favoured particularly by first time mothers, are not small [34,40,42] nor are the potential costs to workplaces/industry for time taken off work to attend to sick children or for GP and hospital appointments [35,43]. This study has focused on costs related to the healthcare sector; future work could include other potential costs, to mothers, their families and the wider society, which are also important to support interventions designed to improve breastfeeding rates.

### Limitations

While the primary care subset represented only 2% of the entire birth cohort, the data source had national coverage, and was a representative, quality assured dataset (i.e., use of standardised codes to describe symptoms and disease). Furthermore, similar patterns of GP consultations have been reported by others; including the rates and types of complaints seen in early childhood [44,45] and the observation of an ‘inverse healthcare law’ for primary care consultations (i.e., higher utilisation and average costs amongst those in the least deprived areas) and a greater use of hospital, including emergency, admissions for infants resident in more deprived areas [46].

Nevertheless, the healthcare costs are likely to be underestimated due to the lower levels of deprivation in the primary care subset compared to the full cohort, the complexity of recording consultations in primary care settings [47] and the decline in complete 6–8 week review visits/incomplete records associated with deprivation [48]. Furthermore, due to limitations in the dataset, the costs did not account for the healthcare provided by other primary healthcare workers (such as community nurses or midwives, health visitors or lactation specialists) or additional details such as the length of consultations or the costs of individual prescriptions. This could be addressed in future research as resources to promote greater use, including the linkage, of primary care data, are developed.

### Policy implications

Child health inequalities, which are both preventable and unfair, remain a priority for public health. Attributed mainly to the unequal distribution of resources—material, psychosocial, cultural and structural–health inequalities operate through a complex web of social stratification, differential exposure and vulnerability across a range of sectors with cumulative effects across the life course [49].

Transforming child health outcomes requires a multifaceted approach that harnesses the potential of parents, carers and communities to provide children with the best possible start in life. Adapting successful lessons from other countries including cultural norms [50], better child welfare policies and enacting more comprehensive paid family leave could help families make informed decisions [51]. Transformations could also comprise more direct measures such as the full implementation of the international code of marketing of breastmilk substitutes [52], improving evidence to address gaps in clinical, community and maternal knowledge/experience—equitably [53]. None of these measures would be without cost but the dividends reaped would be well worth the investment, particularly for the most vulnerable.

## Conclusion

To our knowledge, this is the first population-level birth cohort study to provide a detailed analysis of the direct cost differences in paediatric healthcare associated with different modes of infant feeding in the UK. The benefits of breastfeeding were clear for infants across the spectrum of socio-economic circumstances, including those in the most deprived quintile, as was the ‘extra’ burden of ill health due to formula feeding in both primary and secondary care settings. Ensuring that all infants have the best nutritional start can contribute to reducing inequalities in early childhood.

## Supporting information

S1 File (PDF)

S2 File (DOCX)

S3 File Selected childhood illnesses—ICD 10 and read codes. (DOCX)
