# Supplementary material for: Levelling up health in the early years: A cost-analysis of infant feeding and healthcare
Source: PLoS One. 2024 May 22;19(5):e0300267. doi: 10.1371/journal.pone.0300267 (PMC11111004; doi:10.1371/journal.pone.0300267)
Supplement: S1 File — (PDF) [file pone.0300267.s001.pdf]

Tomi Ajetunmobi

From: Tomi Ajetunmobi
Sent: 11 April 2024 09:17
To: Emma McIntosh; Bruce Whyte; plosone
Cc: David Tappin; Diane Stockton
Subject: RE: ACTION REQUIRED for PONE-D-23-14177

Good morning,
I am happy with this also.

Sincerely,
Omotomilola ('Tomi) Ajetunmobi

From: Emma McIntosh <Emma.McIntosh@glasgow.ac.uk>
Sent: Thursday, April 11, 2024 9:06 AM
To: Bruce Whyte <Bruce.Whyte@glasgow.ac.uk>; plosone <plosone@plos.org>
Cc: David Tappin <David.Tappin@glasgow.ac.uk>; Diane Stockton <Diane.Stockton2@phs.scot>; Tomi Ajetunmobi <Tomi.Ajetunmobi@phs.scot>
Subject: Re: ACTION REQUIRED for PONE-D-23-14177

You don't often get email from emma.mcintosh@glasgow.ac.uk. Learn why this is important

Happy with this.
Emma

Emma McIntosh, PhD *(She/her/hers)*
Professor of Health Economics
School of Health & Wellbeing
College of Medical, Veterinary and Life Sciences
University of Glasgow
Clarice pears building
90 Byres road
Glasgow, G12 8TB

***Follow** us on **X**: @UofGHEHTA*
and @emmasarahmac23

Leading research | Improving health | Tackling inequalities

You are not expected to reply to this email outside of your normal working hours or if you are on leave

---

From: Bruce Whyte <Bruce.Whyte@glasgow.ac.uk>
Date: Thursday, 11 April 2024 at 09:00
To: plosone <plosone@plos.org>
Cc: David Tappin <David.Tappin@glasgow.ac.uk>, Diane Stockton <Diane.Stockton2@phs.scot>, Tomi Ajetunmobi <tomi.ajetunmobi@phs.scot>, Emma McIntosh <Emma.McIntosh@glasgow.ac.uk>
Subject: RE: ACTION REQUIRED for PONE-D-23-14177

I am happy with this also

Bruce Whyte

From: David Tappin <David.Tappin@glasgow.ac.uk>
Sent: Wednesday, April 10, 2024 7:14 PM
To: Diane Stockton <Diane.Stockton2@phs.scot>; plosone <plosone@plos.org>; Tomi Ajetunmobi <tomi.ajetunmobi@phs.scot>; Emma McIntosh <Emma.McIntosh@glasgow.ac.uk>
Cc: Bruce Whyte <Bruce.Whyte@glasgow.ac.uk>
Subject: Re: ACTION REQUIRED for PONE-D-23-14177

I am happy with this.
Thanks
David Tappin
Sent from Outlook for iOS

From: Diane Stockton <Diane.Stockton2@phs.scot>
Sent: Wednesday, April 10, 2024 5:37:31 PM
To: plosone <plosone@plos.org>; Tomi Ajetunmobi <Tomi.Ajetunmobi@phs.scot>; Emma McIntosh <Emma.McIntosh@glasgow.ac.uk>; David Tappin <David.Tappin@glasgow.ac.uk>
Cc: Bruce Whyte <Bruce.Whyte@glasgow.ac.uk>
Subject: RE: ACTION REQUIRED for PONE-D-23-14177

Happy with this.
Thanks
Diane

From: plosone <plosone@plos.org>
Sent: Wednesday, April 10, 2024 5:32 PM
To: Tomi Ajetunmobi <Tomi.Ajetunmobi@phs.scot>; emma.mcintosh@glasgow.ac.uk; Diane Stockton <Diane.Stockton2@phs.scot>; david.tappin@glasgow.ac.uk
Cc: Bruce Whyte <Bruce.Whyte@glasgow.ac.uk>
Subject: ACTION REQUIRED for PONE-D-23-14177

> You don't often get email from plosone@plos.org. Learn why this is important

Dear authors,

We received a request to amend the author list on the PLOS ONE manuscript, "Levelling up health in the early years: A cost-analysis of infant feeding and healthcare" (PONE-D-23-14177). The corresponding author would like to update the byline as follows:

Omotomilola Ajetunmobi
Emma McIntosh
Diane S Stockton
David Tappin
Bruce Whyte

Before I can move forward, I need approval from the following authors:

Omotomilola Ajetunmobi
Emma McIntosh
Diane S Stockton

David Tappin
Bruce Whyte

1. The above authors: Please respond to this email and CC the corresponding author to confirm your agreement to the proposed author list.
2. The corresponding author: Please compile all of the positive responses from the above authors into a single PDF and return this file by email.

I can proceed once the file containing all positive responses has been received. I'm happy to help with any questions, and look forward to your response!

Best,
Nikki Vea Orong
Straive Editorial Assistant

PLOS ONE | plosone@plos.org

Empowering researchers to transform science

Case Number: 08443166
ref:!00DU00Ifis.!500PM0AUobB:ref

This email is intended for the named recipient only. If you have received it by mistake,
please (i) contact the sender by email reply; (ii) delete the email from your system; .
and (iii) do not copy the email or disclose its contents to anyone.
