# Supplementary material for: Levelling up health in the early years: A cost-analysis of infant feeding and healthcare
Source: PLoS One. 2024 May 22;19(5):e0300267. doi: 10.1371/journal.pone.0300267 (PMC11111004; doi:10.1371/journal.pone.0300267)
Supplement: S3 File — (DOCX) [file pone.0300267.s003.docx]

**Supplementary Appendix. Table A: List of codes used to define ill health in cohort (ICD and Read code groups)**

|  | Ill health condition | Diagnostic codes |
| --- | --- | --- |
| Hospital admission (Secondary care  ICD 10 codes - main diagnoses) | Gastrointestinal infections | ‘A02’, ‘A03’, ‘A04’, ‘A05’, ‘A06’, ‘A08’, ‘A09X’, ‘K529’, ‘K521’, ‘K522’, ‘K528’, ‘P783’, ‘R11X’, ‘P920’ |
|  | Upper respiratory tract infections | ‘J00X’, ‘J01’, ‘J02’, ‘J03’, ‘J04’, ‘J05’, ‘J06’,’ ‘J101’, ‘J111’ |
|  | Lower respiratory tract infections | ‘J10’, ‘J11’,’J12’, ‘J13X’, ‘J14X’, ‘J15’, ‘J16’, ‘J17’, ‘J18’,’J20’, ‘J22X’,’R062’,’J100’,’J110’, ‘J40X’, ‘J41’,’J42X’, ‘R05X’ |
|  | Urinary tract infections | ‘N390’, ‘N30’, ‘N34’ |
|  | Otitis media | ‘H65’, ‘H66’, ‘H67’ |
|  | Asthma | ‘J45’, ‘J46X’ |
|  | Allergy | ‘T784’, ‘T781’, ‘T887’, ‘J301’, ‘J302’, ‘J303’, ‘J304’, ‘J450’, ‘L23’, ‘K522’, ‘T780’, ‘T782’, ‘T783’, ‘T886’ |
|  | Eczema | ‘L20’, ‘L21’, ‘L22X’, ‘L23’, ‘L24’, ‘L25’, ‘L26X’, ‘L27’, ‘L28’, ‘L29’, ‘L30’ |
|  | Diabetes | ‘E10’, ‘E11’, ‘E12’, ‘E14’, ‘E14’, ‘P702’ |
|  | Fever | ‘R560’, ‘R509’ |
|  | Dental caries | ‘K029’ |
| Primary care  (Read codes  Scottish version 2) | Gastrointestinal infections | ‘A0’, ‘Ayu0’, ‘A076’, ‘A082’, ‘A0743’, A0740’, ‘A0741’, ‘A083’, ‘19F’, ‘19G’, ‘J433’, ‘J43z’, ‘J432’, ‘Q46y1’, ‘J525’, ‘E2643’, ‘J42’, ‘J422’, ‘J5210’, |
|  | Respiratory infections | ‘A076’, ‘199’, ‘J16y5’, ‘Q4833’, ‘J162’, ‘R0704’, ‘E2754’, ‘A78y1’, ‘E2624’, ‘1998’, ‘R0702’, ‘199’,’J16y5’, ‘Q4833’,’J162’, ‘R0704’, ‘H02..’ H2..’, ‘H50.’, ‘H551.’,’Hyu0’, ‘HyU11’, ‘173e’, ‘R060E’, ‘R060G’, ‘R060F’, ‘R060H’, ‘2326’, ‘173B’, ‘R062’, ‘171’, ‘A33’, ‘E2611’, ‘E1453’, ‘Fyu5F’, ‘R0630’,’H243’, ‘H00’, ‘H07’, ‘1656’ |
|  | Otitis media | ‘A552’, ‘F52’, ‘F51’ |
|  | Allergies | ‘12R’, H1711’, ‘SN58’,’H330’,’D310’, ‘H17’,’14L’,’N062’, ‘J1540’, J0720’, ‘M280’, ‘Sn52’, ‘M128’, ‘M114’, F4D31’, ‘F5130’,’F4C06’, ‘H35’, ‘Zv14’, ‘J432’, ‘ZV150’, ZVu6R’, ‘ZVu6q’, ‘ZVu6i’, ‘ZVu6o’, ‘ZVu6s’ |
|  | Asthma | ‘H33’, ‘14B4’, ‘102’, ‘1J70’, ‘H47y0’, ‘1780’, ‘8H2P’, ‘H3120’, ‘66Yr’, U60F6’, ‘66Yq’ |
|  | Urinary tract infections | ‘K190’ |
